# End of Volume Seven

**Published:** 1867-02

**Authors:** 


### EDITORIAL AND MISCELLANEOUS.

### END OF VOLUME SEVEN.

With this number, the seventh volume of the Atlanta Journal of Medicine and Surgery will be complete. Such as may desire to have the volume bound, will find an ample index, and we take pleasure in informing them that there are facilities in this city for having the work substantially and cheaply done.

This Journal suspended during the war, and by the war: was revived in March last, under circumstances of the greatest embarrassment, without one dollar to pay expenses; without one single exchange or a single subscriber; the material prospects of the country blasted, and individual fortunes swept away. Still unappalled by this array of obstacles, we launched our enterprise upon the sea of fortune, amidst the breakers and adverse winds and currents, but by the blessing of Providence and the kindness of our friends, we have completed another volume.

If the Journal has not been everything our friends expected, or we ourselves could have wished, the explanation is easy. We had no exchanges; no contributors; no money. We were obliged to work at our profession, day and night, and give all our earnings to pay expenses. The attention due to it was necessarily monopolized by other necessities and demands upon us. We have done, not only without comforts, but necessaries even, that the Journal might live and go forth monthly to our brethren. They have had our best endeavors. We hope they are pleased. With the past we are satisfied, because we could do no more. In the future we see much to encourage us, and we do not hestitate to say that with a complete exchange list, and many valuable contributors, we can make the Atlanta Journal of Medicine and Surgery equal to any in the country.

We thank our brethren of the press for their uniform courtesy—our contributors for many able articles—our patrons for their forbearance and punctuality, and our Heavenly Father for his beneficent favor.

The first No. of volume Eight will be issued early in March. We ask our friends to interest themselves for us. Our burden is heavy: they can take much of it from our shoulders. Such as are in arrears will please remit at our risk. Many, like ourselves, have been stripped by the war, and cannot, conveniently, pay in advance. To such we say, send for the Journal and pay when you can make collections.

